# Establishment of a genome editing tool using CRISPR-Cas9 ribonucleoprotein complexes in the non-model plant pathogen *Sphaerulina musiva*

**DOI:** 10.3389/fgeed.2023.1110279

**Published:** 2023-07-21

**Authors:** Joanna Tannous, Cole Sawyer, Md Mahmudul Hassan, Jesse L. Labbe, Carrie Eckert

**Affiliations:** ^1^ Biosciences Division, Oak Ridge National Laboratory, Oak Ridge, TN, United States; ^2^ Graduate School of Genome Science and Technology, University of Tennessee, Knoxville, TN, United States; ^3^ Department of Genetics and Plant Breeding, Patuakhali Science and Technology University, Patuakhali, Bangladesh

**Keywords:** CRISPR-Cas9, RNP, spore-bearing fungi, ascomycetes, trichoderma reesei, Sphaerulina musiva

## Abstract

CRISPR-Cas9 is a versatile genome editing system widely used since 2013 to introduce site-specific modifications into the genomes of model and non-model species. This technology is used in various applications, from gene knock-outs, knock-ins, and over-expressions to more precise changes, such as the introduction of nucleotides at a targeted locus. CRISPR-Cas9 has been demonstrated to be easy to establish in new species and highly efficient and specific compared to previous gene editing strategies such as Zinc finger nucleases and transcription activator-like effector nucleases. Grand challenges for emerging CRISPR-Cas9 tools in filamentous fungi are developing efficient transformation methods for non-model organisms. In this paper, we have leveraged the establishment of CRISPR-Cas9 genome editing tool that relies on Cas9/sgRNA ribonucleoprotein complexes (RNPs) in the model species *Trichoderma reesei* and developed the first protocol to efficiently transform the non-model species, *Sphaerulina musiva.* This fungal pathogen constitutes a real threat to the genus *Populus,* a foundational bioenergy crop used for biofuel production. Herein, we highlight the general considerations to design sgRNAs and their computational validation. We also describe the use of isolated protoplasts to deliver the CRISPR-Cas9 RNP components in both species and the screening for targeted genome editing events. The development of engineering tools in *S. musiva* can be used for studying genes involved in diverse processes such as secondary metabolism, establishment, and pathogenicity, among many others, but also for developing genetic mitigation approaches. The approach described here provides guidance for potential development of transformation systems in other non-model spore-bearing ascomycetes.

## Introduction

Fungi are often overlooked in nature although their roles in biology, their influence on the environment and their usage across multiple sectors, including industry and medicine are undeniable (Neglecting Fungi, 2017). These organisms play essential roles in ecosystems by being the earth’s best degraders of organic matter and providing nutrients critical for plant growth (Frac et al., 2018). In addition, fungi’s greatest attributes are their ability to produce industrial enzymes and secondary metabolites, which hold prodigious potential for biotechnological and pharmaceutical applications (Keller, 2019; Shankar and Sharma, 2022). Yet, besides these beneficial functions some fungal species are known to be pathogenic and cause life-threatening diseases or significant reduction in agricultural activities and food production worldwide (Doehlemann et al., 2017; Fausto et al., 2019; Rokas, 2022). The development of tools to genetically modify these organisms is inevitable to improve the implementation of their benefits or reduce their negative impacts.

Ascomycota is the largest fungal phylum, with well over 93,000 described species, consisting of soilborne saprotrophs, opportunistic human pathogens or plant pathogens and symbionts. (Schoch et al., 2009). This phylum encompasses both yeasts and filamentous fungal morphologies. Transformation efficacy is low in filamentous fungi compared to yeasts, mainly due to their rigid cell wall, and therefore it is considered as a holdup in genetic engineering (Ruiz-Díez, 2002). Different genetic transformation systems have been developed in a variety of filamentous fungi. Physical gene editing methods such as biolistics (also known as particle bombardment), glass bead method, and electroporation have been optimized in *Trichoderma harzianum*, *Trichoderma reesei* (Te’o, et al., 2002), *Blumeria graminis* (Christiansen et al., 1995)*, Aspergillus niger* (Ozeki et al., 1994)*, Aspergillus nidulans* (Fungaro et al.,. 1995; Sánchez and Aguirre, 1996)*,* and *Fusarium oxysporum* (Singh, 2012) among others. Chemical transformation systems that rely on the use of chemicals that enhance membrane permeability and DNA uptake, such as calcium chloride or lithium acetate, have been developed in *A. nidulans* (Tilburn et al., 1983) and *Neurospora crassa* (Paietta and Marzluf, 1984). Lastly, biological systems that employ the use of *Agrobacterium tumefaciens* for transformation have been successfully established in *Aspergillus awamori* (Gouka et al., 1999), *Fusarium venenatum* and others (Groot et al., 1998).

The utmost shift to the field of genome editing in filamentous fungi was the discovery and development of the CRISPR-Cas9 system that enables the targeted cleavage of a specific double-stranded DNA sequence by an RNA-guided endonuclease in a precise, efficient, and highly versatile mode (Mali et al., 2013). The Cas9 enzyme can be delivered into the host cell in three different forms, either DNA, mRNA, or protein, depending on the organism and the transformation approach being used, which enables good adaptability. Each approach has its own characteristics, prerequisites, benefits, and downsides (Lin et al., 2022). When introduced in DNA form, the primary advantage is that a single construct that harbors all the necessary components for editing (a *Cas9* gene under constitutive expression, a selectable marker, a single-guide RNA (sgRNA) expression cassette, and a donor DNA (dDNA) sequence) can be successfully transformed into the cell. However, the use of this form is dependent on the availability of an expression system for the species of interest (Wilson and Wingfield, 2020). The RNA-based delivery of the CRISPR/Cas9 system also comes with challenges, mainly due to the instability of the RNA during the transformation process (Wilson and Wingfield, 2020). On the other hand, the ribonucleoprotein (RNP)-mediated editing has become an attractive method offering many benefits over the two other techniques. The ease of design, effectiveness, and adaptability enable the use of this approach in various biological systems, particularly in organisms without a well-established genetic manipulation system. Moreover, RNPs provide a shorter exposure time of the targeted genome to active Cas9 enzymes, which reduces the risk of off-target editing (S. Kim et al., 2014).

Another major benefit of using the CRISPR-Cas9 system is the ability to edit multiple genes simultaneously, allowing to create mutants with multiple site mutations in a single transformation (Jiang et al., 2021). The CRISPR-Cas9-DNA double strand breaks can be repaired through either the nonhomologous end joining (NHEJ), which is the main repair pathway or by homology-directed repair (HDR). The latter can be used to introduce specific sequences into the gRNA cutting site if exogenous donor DNA is provided, whereas NHEJ typically engenders short insertion/deletion (indels) or frameshift mutations at the repair junctions leading to premature stop codons within the open reading frame (ORF) of the targeted gene (H. Kim and Kim, 2014). Various versions of CRISPR/Cas9 tools have been developed in filamentous ascomycete fungi and each of these methods may be the preferred approach for a specific species. The model species *T. reesei* was the first filamentous ascomycete fungus in which a CRISPR/Cas9 editing tool was applied (Liu et al., 2015). Since then, this editing system has found wide applications in other ascomycete filamentous fungal genera, such as Neurospora (Matsu-ura et al., 2015), *Aspergillus* (Katayama et al., 2016; Abdallah et al., 2017), *Penicillium* (Pohl et al., 2016; Grijseels et al., 2018), and *Fusarium* (Gardiner and Kemal, 2018; Shi et al., 2019).

*Sphaerulina musiva* (formerly known as *Septoria musiva*), a member of the Mycosphaerellaceae family, is an ascomycete plant pathogen often encountered on *Populus* plantations and responsible of necrotic leaf spots and stem canker diseases on susceptible plant genotypes (Feau et al., 2010). While high quality genomic and transcriptomic data are available for this species (Tabima et al., 2020), no genome editing protocols have been developed, and hence the research to decipher the mechanisms underlying the *S. musiva*- *Populus* pathosystem has been mainly focusing on the host side (Liang et al., 2002; Dunnell and Leboldus, 2017; Abraham et al., 2019; Lenz et al., 2021; Kelsey L.). This paper describes the development of the first transformation system for *S. musiva* using a protein-based version of the CRISPR-Cas9 genome editing system. Herein, we target the *pyrG* gene encoding for Orotidine 5′-phosphate decarboxylase that confers prototrophy to uracil and uridine. This approach was developed after validation of the CRISPR/Cas9 gene editing in RNP format in the model ascomycete fungal species *T. reesei.* The initial exploitation of this system in a model organism using pre-validated sgRNAs is important and aims to validate the feasibility of successfully establishing an RNP version of the CRISPR-Cas9 genome editing system in filamentous fungi.

## Methods

### Organisms and targeted genes

*Trichoderma reesei—T. reesei* strain QM6a (ATCC13631) (Li et al., 2017) was used in this study. The targeted gene is the *ura5* auxotrophy gene.

*Sphaerulina musiva*—*S. musiva* isolate MN14 was used in this study to establish the CRISPR-Cas9 RNP-mediated transformation. This species was isolated from canker of an interspecific hybrid poplar (*P. nigra x P. maximowiczii*) tree in Minnesota, United States (Tabima et al., 2020). The gene targeted in this transformation is the *pyrG* encoding for Orotidine 5′-phosphate decarboxylase that confers prototrophy to uracil and uridine.

### Design and computational validation of sgRNAs

*Trichoderma reesei—*To ensure the successful development of the CRISPR-Cas9 RNP-mediated transformation system, we tested the gRNA (5ʹ-GGA​TGC​CGA​AAT​CAT​GGC​CGt​gg-3ʹ, PAM shown in lowercase) designed and confirmed in the study by Liu et al. (2015) that targets the *ura5* gene in the model fungus *T. reesei.*

*Sphaerulina musiva*—In addition to the Cas9 nuclease, the CRISPR-Cas9 gene editing requires a custom single guide RNA (sgRNA) that is made up of two parts: i) the Crispr RNA (crRNA) that comprises 20 nucleotide sequence complementary to a region in the target gene that will direct the Cas9 nuclease activity and ii) the trans-activating crRNA (tracrRNA) that serves as a binding scaffold for the Cas9 nuclease. First, we run a Blast search using the *pyrG* gene from *A. nidulans* FGSC A4 (XM_658,669.1) against the *S. musiva* strain SO2202 genome on NCBI. A local blastn search against the genome of strain MN14 was later performed to locate the coordinates of *pyrG* in the isolate of interest. For the design of the crRNA, the web tool CRISPOR (http://crispor.tefor.net/) is used. This tool lists possible crRNA sequences within a specific DNA region and provides scores such as specificity, efficiency prediction, out of frame, and off targets. To start generating crRNA sequences, copy and paste the sequence of the target gene, and select the genome of interest from their list. If the genome does not exist in the list, the GCF_/GCA_ ID from NCBI can be sent to the CRISPOR support team. Lastly, we selected a Protospacer Adjacent Motif (PAM). For our analysis, the gene of interest was the Orotidine 5′-phosphate decarboxylase encoding gene (*pyrG*) from *Sphaerulina musiva* strain MN14 and the selected PAM is -NGG specific for *Streptococcus pyogenes* (Sp)-derived Cas9 (SpCas9). Below are the different criteria we followed in the selection of the crRNA sequence.

⁃ It is recommended to select from the list gRNAs that target conserved coding domains of the gene of interest.
⁃ The MIT specificity score summarizes all off targets into a single number from 0–100. The higher the number, the less off-target effects are predicted. It is recommended that crRNA have an MIT specificity score >50.
⁃ The Cutting Frequency Determination (CFD) specificity score is a prediction of the interaction between the guide and the target. The values range between 0 and 100, with 0 being the weakest interaction due to mismatches between the guide and the DNA target.
⁃ The Doench efficiency score is a prediction of the efficiency of cutting and is recommended to be > 60.
⁃ The off-target mismatch counts represent the number of possible off-targets in the genome, for each number of mismatches. This is a summary of the whole-genome search for sequences similar to the guide target sequence.
⁃  The out of frame score corresponds to the probability that mutations induced by the gRNA will disrupt the open reading frame.

After selecting the crRNA sequence with the best location, high predicted activity score and low off-target scores, we used the RNAfold webserver (http://rna.tbi.univie.ac.at/cgi-bin/RNAWebSuite/RNAfold.cgi) to ensure that the RNA molecule will fold into the correct 3D structure for binding to the Cas9 enzyme. We generated a combined sequence including the protospacer and the Cas9-specific scaffold sequence and proceed to secondary structure prediction on RNAfold. For our analysis the gRNA scaffold sequence used is: GTT​TTA​GAG​CTA​GAA​ATA​GCA​AGT​TAA​AAT​AAG​GCT​AGT​CCG​TTA​TCA​ACT​TGA​AAA​AGT​GGC​ACC​GAG​TCG​GTG​C (Liu et al., 2015).

Lastly, we evaluated the RNAfold results and chose a final crRNA candidate from those that passed the different filtering steps. In this regard, it is recommended that the crRNA and tracrRNA exhibit interactions without affecting the essential stem-loop structures of the sgRNA. The secondary structure of the sgRNA should contain three stem loops, interrupted by five ring structures. High binding probabilities should also be displayed through the entire structure, except for the protospacer region.

### Preparation of sgRNA-Cas9 ribonucleoprotein complex

The designed crRNA, tracrRNA (Cat No. 1072533) and the Cas9 nuclease (Cat No. 1081059) were synthesized by Integrated DNA Technologies (IDT). First, we diluted the crRNA and tracrRNA to a final concentration of 200 µM in IDT duplex buffer (Cat No. 1072570). To prepare the sgRNA, we mixed 5 µl of 200 µM crRNA and 5 µl of 200 µM tracrRNA and completed the volume to 20 µl with IDT duplex buffer. The sgRNA mixture was later heated to 95°C for 5 min and allowed to cool down at room temperature for at least 20 min. Lastly, to assemble the Cas9/sgRNA ribonucleoprotein (RNP) complex, we mixed 6 µl of sgRNA and 8.3 µl of the Cas9 nuclease (60 µM stock) and completed the volume to 25 µl using RNase-free water.

### Assessing the cleavage ability of the S. musiva sgRNA *in vitro*

This step is optional but recommended to test the ability of the sgRNA to cleave *in vitro*. First, we designed a primer set (Smusiva_pyrG F/R) that will amplify a fragment harboring the chosen sgRNA target site. Genomic DNA from *S. musiva* strain MN14 was extracted following the protocol from Tannous et al. (2018). The targeted DNA fragment was amplified using the designed primer set and a standard DNA polymerase following the manufacturer instructions. We later prepared the sgRNA by combing 0.5 µl of the 200 µM crRNA and 0.5 µl of the 200 µM tracrRNA from the step above into a final volume of 10 µl duplex buffer. The mixture was incubated at 95°C for 5 min, then allow it to cool at room temperature. To form the RNP complex, we combined the 10 µl of sgRNA with 1.6 µl of Cas9 (60 µM stock) and completed the volume to 100 µl with Cas9 dilution buffer (30 mM HEPES, 150 mM KCI, pH 7.5). The mixture was incubated at room temperature for 10 min for optimal formation of the RNP complex. The *in vitro* digestion reaction was performed by combining 1 µl of the Cas9 dilution buffer, 1 µl of the Cas9-RNP complex, 3 µl of the purified PCR product into a final volume of 10 µl with nuclease free water. The reaction was incubated at 37°C for 60 min. To release the DNA substrate from the Cas9 nuclease, we added 2 μl of Proteinase K (10 mg/mL) to the reaction, then incubated the mixture at 56°C for 10 min. Lastly, the digestion reaction and the PCR product were analyzed on a 2% agarose gel to visualize the cleaved products.

### Isolation of protoplasts and PEG-mediated transformation

**FIGURE 1:**
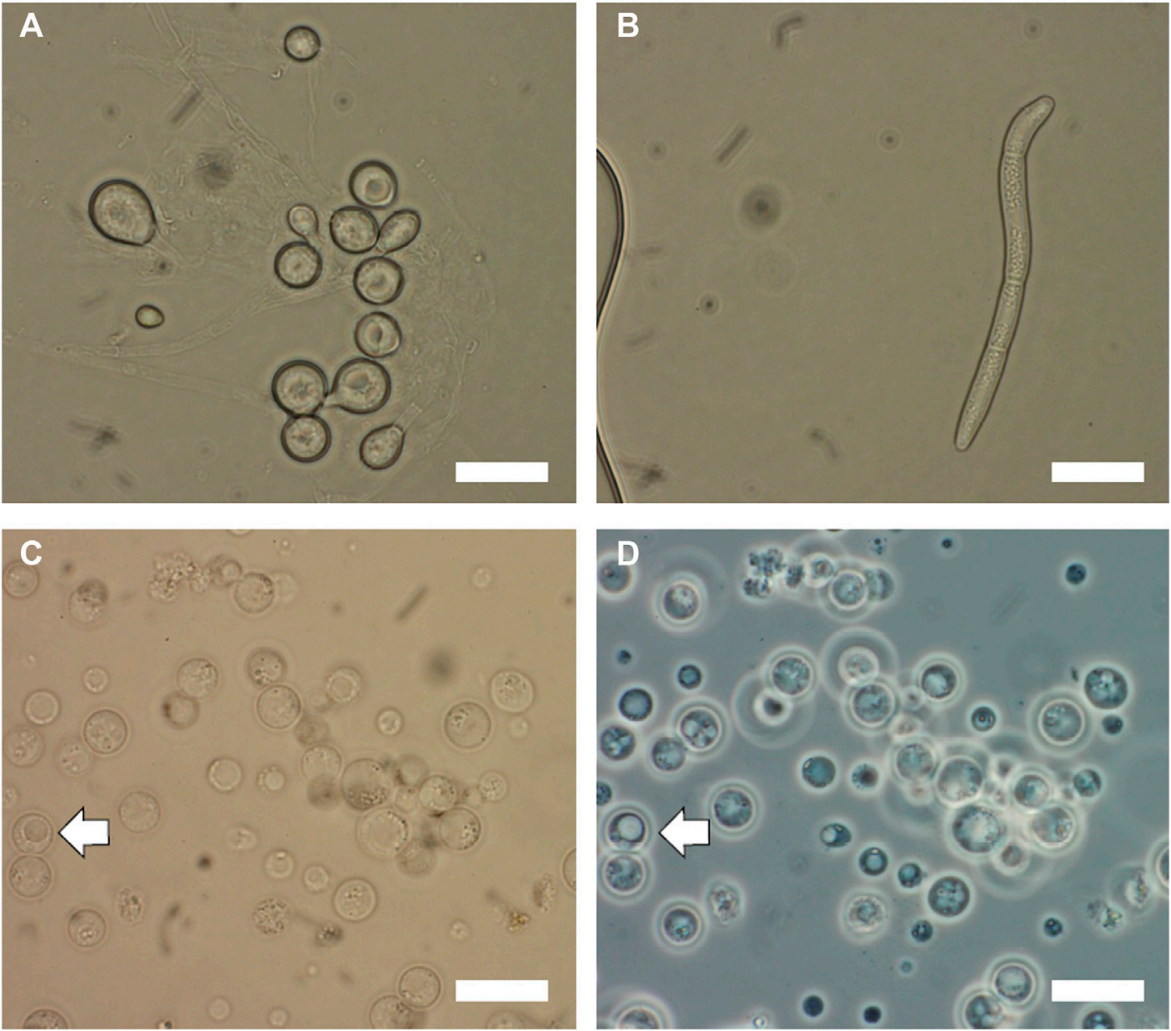
Microscopic images of *Trichoderma reesei* and *Sphaerulina musiva* spores and protoplasts. Brightfield microscopy optics of **(A)** *T. reesei* spores collected from 48 h cultures on PDA medium at 25°C and **(B)** *S. musiva* spores collected from 5 days cultures on KV8 medium at 21°C. Images of *S. musiva* isolated protoplasts after 3 h enzymatic digestion using **(C)** brightfield optics and **(D)** phase contrast optics. White arrow indicates protoplast with vacuole. Scar bars are 10 μm.

First, the cryo-preserved spores of *T. reesei* and *S. musiva* were activated on PDA and KV8 agar medium (180 ml V8 vegetable juice, 2 g CaCO_3_, 20 g agar in 1 L distilled water, pH = 7–7.2) supplemented with streptomycin (100 μg/ml), respectively. The plates were incubated for at least 5 days at 25°C. To isolate protoplasts from fungal cells, we inoculated 10^8^ fresh spores of each of the fungal species collected using sterile water. The morphology of spores collected from both species are shown in Figures 1A, B Depending on the fungal species, the incubation time prior to protoplasting can vary. For *T. reesei*, 24 h growth were required to get enough fungal biomass, whereas for *Sphaerulina musiva* which is a slow grower species, we allowed 72 h of growth prior to protoplasting. To harvest the germlings (or germinated spores), we transferred the liquid culture into 50 mL falcon tubes and centrifuged at 4,000 *g* for 5 min. The supernatant was discarded, the germlings were washed with sterile water to eliminate all traces of media and centrifuged again to collect the germling pellet. To lyse the cell wall of the young germlings and release protoplasts, the germling pellet was resuspended in 10 ml of protoplasting solution (100 mg of lysing enzyme from *T. harzianum* and 50 mg of yatalase in 10 ml osmotic medium (1.2 M MgSO_4_ and 10 mM Sodium Phosphate Buffer). The protoplasting solution/germlings preparation was incubated at 30°C with shaking at 80 rpm for 3–4 h. The preparation should be checked every hour for protoplast release. Protoplasts will appear as shown in Figure 1C, d after complete cell wall digestion. Protoplasts are cells without cell walls and are thus very sensitive to mechanical disruption. Make sure to handle them gently when pipetting. To collect protoplasts, we overlayed the preparation gently with 10 ml of cold Trapping buffer (0.6 M Sorbitol and 0.1 M Tris-HCl, pH 7), and centrifuged at 5,000 *g* and 4°C for 15 min. The protoplasts were collected from the interface into a new 15 ml centrifuge tube and overlayed with an equal volume of cold STC buffer (1.2 M Sorbitol, 10 mM CaCl_2_, 10 mM Tris-HCl, pH 7.5). The protoplasts were later pelleted by centrifuge at 3,000 *g* and 4°C for 10 min. To initiate the transformation, we combined 100 µL of isolated protoplasts with 25 µl of the RNP complex mixture and completed the volume to 200 µL using STC buffer. We vortexed the solution gently to ensure it is thoroughly mixed. A negative control should be prepared by mixing 100 µL of isolated protoplasts with 100 µL of STC buffer. The transformation mixture and the negative control were incubated on ice for 50 min. Later, 1.2 mL of PEG were added to both mixtures and incubated for an additional 20 min at room temperature. Then, we added 5 mL of STC buffer to both mixtures and gently homogenized them. After incubation, we combined the transformation mixture with 45 mL of molten Sorbitol Minimal Medium (SMM) top agar medium (SMM with reduced amount of agar by half) (Tannous et al., 2023) supplemented with uracil (0.56 g/L) and uridine (1.26 g/L) and poured 5 mL of the mixture on top of plates containing 20 mL of SMM supplemented with the same amino acids. For the negative control, we combined 1 mL of the mixture with 5 mL of Top agar medium and poured on top of SMM medium plates supplemented with the same amino acids. The first layer of top agar was allowed to set by incubating the cultures overnight at 21°C (for *S. musiva*) and 25°C (for *T. reesei*). The next day, we added an additional layer of SMM top agar supplemented with 5- Fluoroorotic acid (5-FOA) at a final concentration of 1.5 mg/ml of medium. The cultures were incubated again until single transformants were observed growing through both layers of agar. To check the stability of the protoplasts through the transformation process, we recommend having a positive control plate not supplemented with 5-FOA.

Individual isolates were later picked and screened on new SMM plates supplemented with 5-FOA and uracil and uridine. Lastly, we extracted gDNA from all selected *T. reesei* and *S. musiva* transformants using the method cited above.

### Screening for genome editing events

To screen for the genome editing events in *T. reesei*, we designed the primer set (Treesei_Ura5 F/R) that will amplify fragment of the *ura5* gene harboring the sgRNAs target sites. For *S. musiva*, we used the same set of primer designed for the *in vitro* validation of the gRNA cleavage to amplify the fragment harboring the gRNA cut site. The primer sequences are presented in Table 1. We amplified the two fragments using a standard DNA polymerase and purified the PCR products prior to sequencing using the QIAquick PCR Purification kit (Qiagen, Cat. No. 28104). The sequences from the different transformants were lastly aligned with the WT sequence of the gene for INDEL detection at the gRNA cut site.

**TABLE 1:** Primer pairs used in this study to confirm the gene editing events.

Primer name	Sequence (5′-3′)
Treesei_Ura5 F	GCG​GCG​TCC​TCA​AGT​TTG​GC
Treesei_Ura5 R	CGG​TAA​TCC​TCC​GTG​TTC​TT
Smusiva_pyrG F	CTG​GAA​GGA​AGA​CGG​ACA​GA
Smusiva_pyrG R	GAT​GGC​TTT​CTG​TGC​AGC​TT

## Results

### Computational and *in vitro* validation of the designed sgRNA targeting the pyrG gene in S. musiva

**FIGURE 2:**
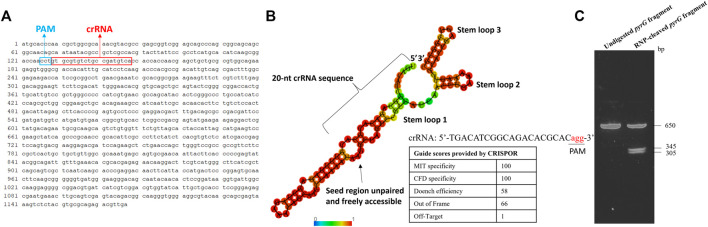
*In silico* and *in vitro* analyses of the gRNA targeting the *pyrG* gene in *S. musiva* strain MN14*.* **(A)** Sequence of the *S. musiva* strain MN14 *pyrG* gene showing the location of the designed crRNA **(B)**. *In silico* analysis of gRNA secondary structure to predict its efficacy for genome editing in *S. musiva*. The crRNA and tracrRNA exhibit marginal interactions not affecting the essential stem-loop structures of the sgRNA. The RNA secondary structure was predicted using the RNAfold web server (http://rna.tbi.univie.ac.at/cgi-bin/RNAWebSuite/RNAfold.cgi). The color scale displays the base-pairing probability. The table shows the scoring algorithms provided by the web-based tool CRISPOR webserver (http://crispor.tefor.net/) for the selected guide. **(C)**. Agarose gel displaying the cleavage of the *pyrG* fragment with the Cas9 RNP complex *in vitro*. The DNA fragment was not fully digested and therefore the 650 bp band is still showing on the gel after digestion.

The approach described in this study employed an RNP version of the CRISPR-Cas9 genome editing system and the critical step in this protocol is the design and synthesis of a high quality sgRNA. The location of the designed crRNA in the *pyrG* sequence of *S. musiva* strain MN14 is shown on Figure 2A. Figure 2B displays the scores of the crRNA sequence provided by CRISPOR. This molecule was selected in such a way that it: i) specifically targets the *pyrG* gene (with an MIT specificity score of 100), ii) shows high interaction score with the target sequence (with a CFD specificity score of 100), iii) is capable of effectively cleave the target region (with a Doench score of 58), iv) shows slight similarity to other regions in the genome (with 1 off target predicted), and v) folds correctly in order to bind with the Cas9 protein. The ability of this sgRNA to target and cleave the target region was later confirmed *in vitro* by showing two products of the expected sizes (Figure 2C).

### Confirmation of the genome editing events in T. reesei and S. musiva

**FIGURE 3:**
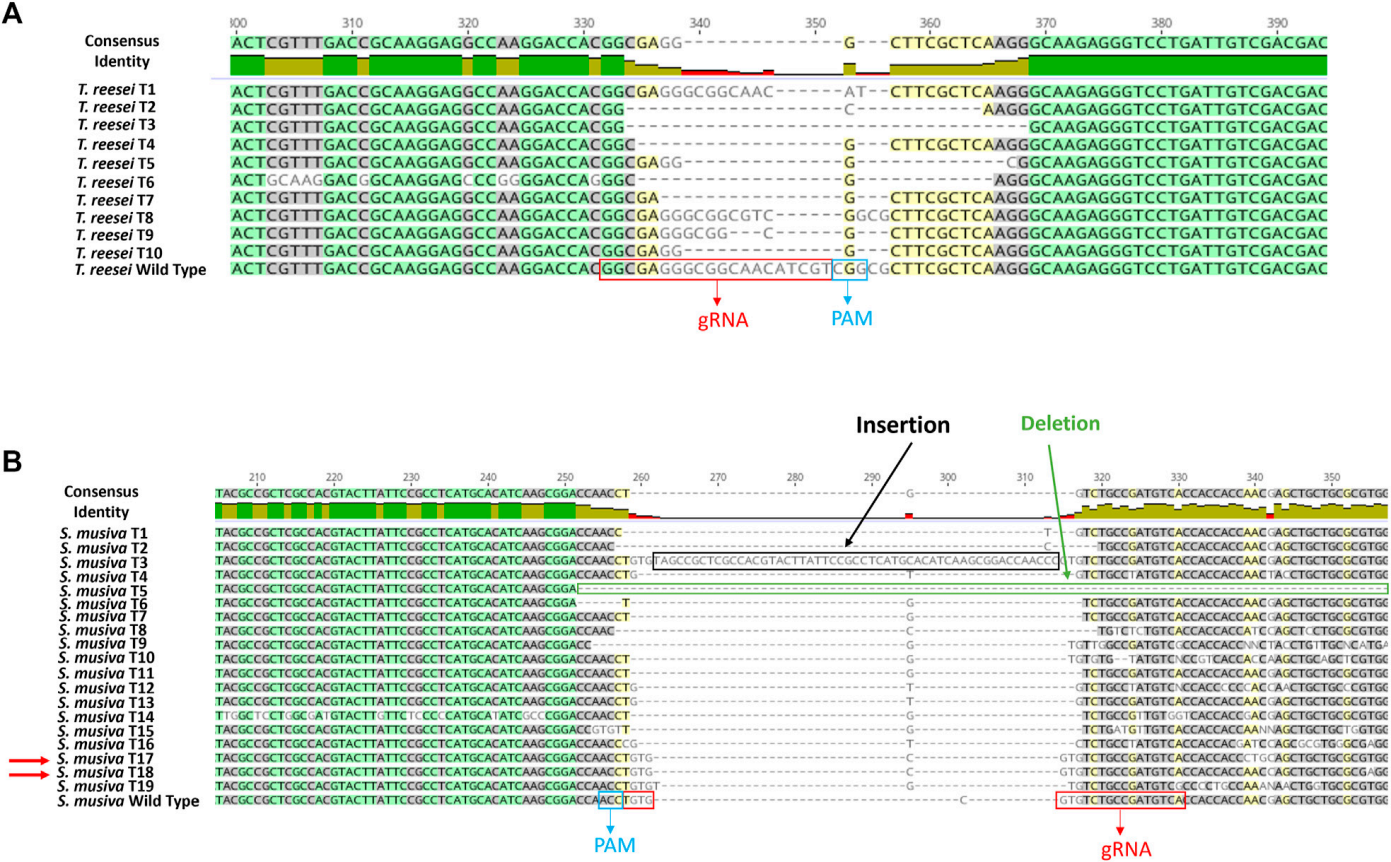
DNA sequence alignment of wild type and mutant mutation site in *Trichoderma reesei* **(A)** and *S. musiva* **(B)**. PCR products from *ura5* and *pyrG* KO strains of *Trichoderma reesei* and *S. musiva*, respectively, were sequenced and aligned with the WT gene sequences using Geneious Prime version 2020.2.2. The gRNA location and the PAM region are showed in red and blue boxes, respectively. Double-strand DNA breaks were repaired by NHEJ generating random insertions and deletions.

For both species, one successful transformation was performed. Ten random transformants were selected from the *ura5*-targeted transformation in *T. reesei.* All 10 selected transformants presented random deletions at the gRNA cut site as shown in the alignment with the WT sequence of this gene in Figure 3B. For this transformation, we used the same gRNA designed by Liu et al. (2015). The difference between the transformation methods was the format of the CRISPR components. In the previous study they were introduced to the cells as mRNA (*in vitro* transcribed) format, whereas here the Cas9 and the sgRNA were delivered as RNP complex. Regardless of the delivery format, we recorded a similar transformation efficiency of 100%.

For *S. musiva*, since this is the first CRISPR-cas9 editing protocol developed on this species*,* 20 transformants were randomly picked and subjected to a second screening on new SMM plates supplemented with 5-fluoroortic acid (5-FOA) and uracil and uridine. The disruption of the *pyrG* gene allows an easy selection on media supplemented with 5-FOA, a chemical that can be converted into the toxic intermediate 5-fluorodeoxyuridine through *pyrG* activity, thus generating *S. musiva* △*pyrG* mutant strains that display auxotrophy for uracil and uridine but insensitivity to 5-FOA.Of the 20 transformants, 19 were able to grow on the screening plates. An amplification and sequencing of the *pyrG* region harboring the gRNA cut site from these transformants and their alignment with the WT *pyrG* sequence showed various indels at the gRNA cut site in 17 isolates (Figure 3B). Two isolates *S. musiva* T17 and T18 showed no differences in the sequence while aligned with the WT copy of the *pyrG* gene. To summarize, a 100% editing efficiency was observed in *T. reesei,* while 90% editing efficiency was observed following *S. musiva*’s transformation.

## Discussion

In this paper we describe the development of a first protoplast-mediated transformation system in the poplar pathogen, *S. musiva*. This was attained via a protein-based version of the CRISPR-Cas9 genome editing system. The method entailed the *in vitro* preparation of the sgRNA, and its assembly with a commercially available Cas9 endonuclease to form RNP complexes used to transform protoplasts extracted from *S. musiva*. The success of this approach was demonstrated by the generation of various indels near the gRNA cut site in the *pyrG* sequence in 90% of the selected transformants (Figure 3B).

**FIGURE 4:**
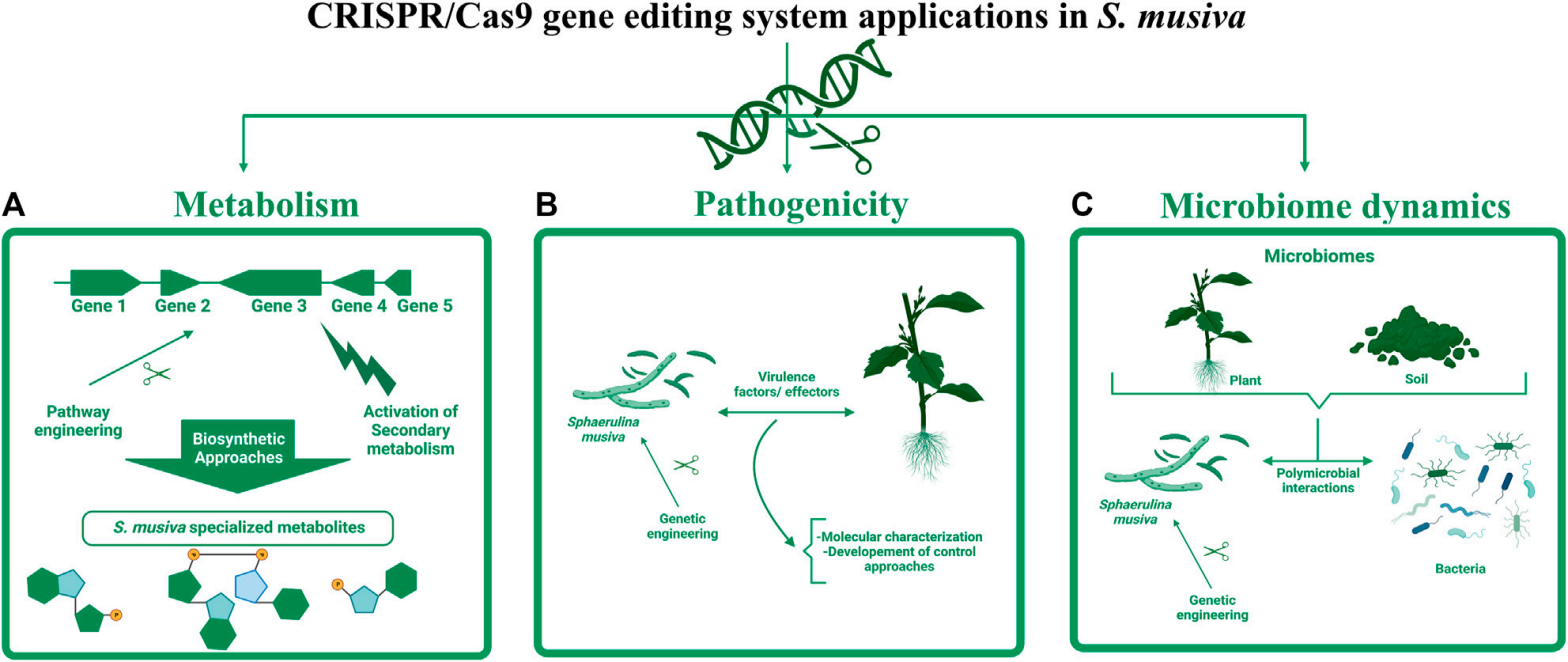
Potential applications of CRISPR-Cas9 editing tool in *S. musiva.* The engineering of this fungal plant pathogen can have various applications ranging from: **(A)** transcriptional activation of silent biosynthetic gene clusters to characterize unknown specialized metabolites, **(B)** identification and characterization of virulence factors and effectors playing important role in *S. musiva*-plant interactions, which can be potential targets for development of genetic mitigation approaches, and **(C)** elucidation of the role of *S. musiva* in the regulation of plant and soil microbiome.

The development of this gene editing tool in *S. musiva* can have various applications ranging from elucidating its metabolome and re-designing metabolic processes, to deciphering its pathogenicity determinants and understanding its role in plant and root microbiomes (Figure 4). In regards of metabolomics, a secondary metabolite biosynthetic gene cluster (BGC) analysis of *S. musiva* carried out by antiSMASH fungal 6.0 (Blin et al., 2019) identifies 31 putative BGCs. Of these, the function of only few clusters is predicted, but has not been experimentally proven, for example, the melanin-like gene cluster (Foster et al., 2014). The developed transformation system will enable the elucidation of metabolite biosynthesis in *S. musiva* by combining gene engineering methods with metabolite analysis since minor variations in gene expression and protein synthesis will potentially result in an amplified modification in the metabolite profile.

Aside from the limited knowledge about *S. musiva*’s metabolome that might be playing critical role in the interaction of this pathogen with its host and surrounding microbes, up to now, little is known about the pathogenicity determinants in this species. A single transcriptomic study was conducted on *S. musiva* during infection of *Populus* susceptible genotypes (Dunnell, 2016). Data from this Ph.D. research allowed the identification of over 100 genes that were upregulated at 3 weeks post-inoculation in the infected trees compared to the control. Most of these differentially expressed genes were hypothetical proteins, with no known function. Yet, no further investigations were conducted to characterize these genes and establish their role in the *S. musiva- Populus* pathosystem due to the lack of a transformation protocol in this species. The development of the CRISPR-Cas9 editing system in *S. musiva* offers a great opportunity to characterize effectors and virulence factors in this pathogen. Lastly, this tool can also be leveraged for the establishment of genetic mitigation approaches that target these virulence factors to reduce diseases on *Populus* and other hosts and ensure sustainable agriculture.

## Data Availability

The original contributions presented in the study are included in the article, further inquiries can be directed to the corresponding authors.
